# 296. Multiple and changing strains of MRSA in bone and joint infections: the EMOJI study

**DOI:** 10.1093/ofid/ofaf695.098

**Published:** 2026-01-11

**Authors:** Eleonora Cella, Kevin Bouiller, Natasia Jacko, Maeve Hiehle, Taj Azarian, Michael Z David

**Affiliations:** University of Central Florida, Orlanda, Florida; Université Marie et Louis Pasteur, Laboratoire Chrono-environnement UMR 6249, Besancon, Bourgogne, France; Division of Infectious Diseases, University of Pennsylvania, Philadelphia, Pennsylvania; University of Pennsylvania, Philadelphia, Pennsylvania; University of Central Florida, Orlanda, Florida; University of Pennsylvania Perelman School of Medicine, Philadelphia, Pennsylvania

## Abstract

**Background:**

*Staphylococcus aureus* (*SA*) is the most common pathogen in bone and joint infections (BJIs). Genetic evolution of BJI isolates during recurrence and/or persistent infection is not well characterized. We investigated the genomic epidemiology of methicillin-resistant *SA* (MRSA) among patients with >1 different BJI isolates. We assessed whether isolates from distinct BJI episodes in a patient belonged to the same intrasubject lineage (ISL).

**Figure:**
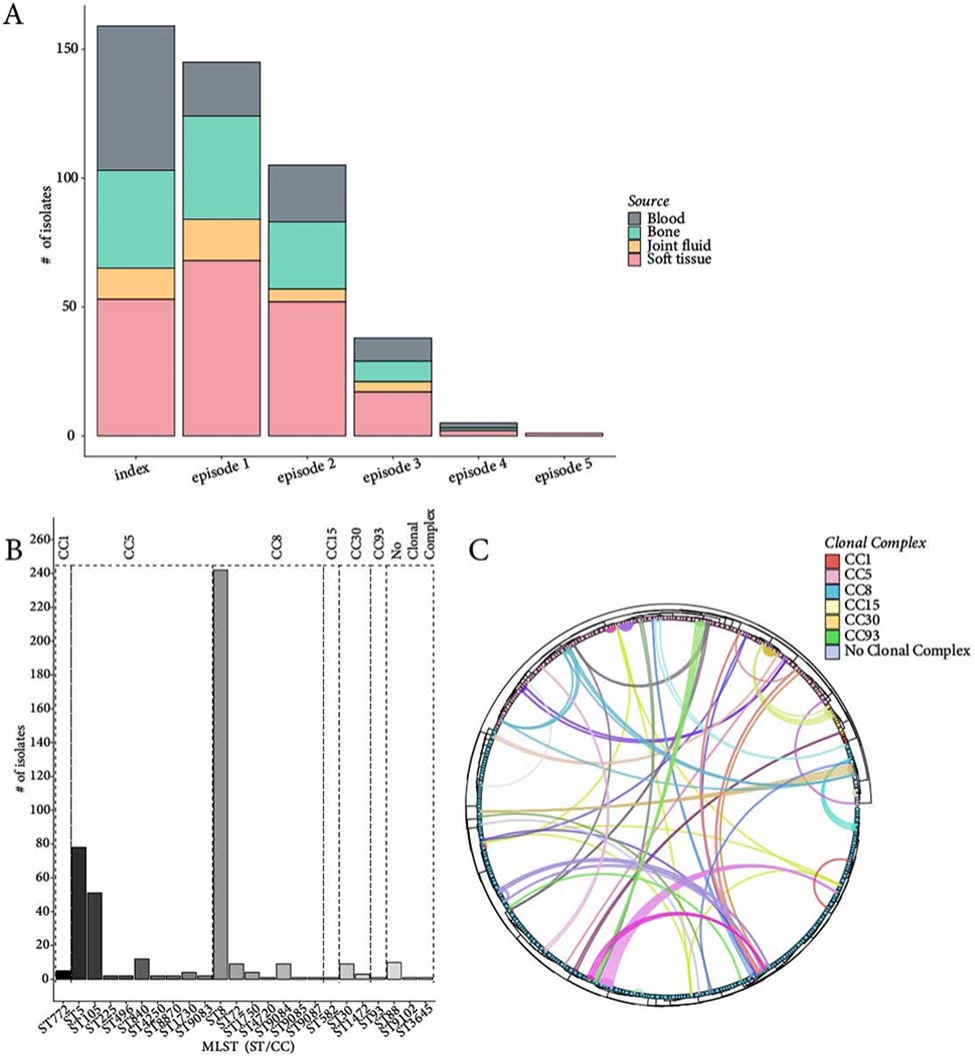
A. Isolate distribution by episode type and by source. B. Staphylococcus aureus lineage of isolates. Number of isolates belonging to each clonal complex (CC), and to each multi-locus sequence type (MLST) within each clonal complex is shown. C. Circos plot of maximum-likelihood (ML) phylogeny and the relationship between co-carried SA strains. A ML phylogeny was generated from a core-SNP alignment of all 453 isolates. Each tip corresponds to an isolate colored according to the clonal complex (legend on the top right). Inside the tree, connections are drawn between isolates that belong to the same patient who shared multiple ISLs; each patient is represented by a unique color.

**Methods:**

All patients with at least 2 MRSA BJI isolates from July 2018 to December 2022 from 2 US academic hospitals were included. Only one isolate per day from the same site (blood, bone, or other deep tissue) was included. The index isolate was defined as the first BJI isolate, and episodes were defined as all isolates collected within 14 days. Episodes were classified as recurrent if they 1) involved a new anatomic site or 2) the same site after a minimum of 6 months without recent antibiotic use or as relapse if they occurred at the same site after 14 days of antibiotics. Isolates underwent sequencing, and phylogenetic analysis was performed.

**Results:**

We enrolled 159 patients who experienced 278 epidemiologically defined BJI episodes; 74 patients had index episodes only and 85 had a range of 2-5 episodes spanning 1-1,403 days (mean=133). Subsequent episodes were categorized as 74 new infections and 45 recurrences (48.9% involving a new site). The 453 sequenced isolates were classified by episode type and source (Fig. 1A). Soft tissue was the most common source of isolation followed by blood and bone. Population structure analysis further resolved the MRSA isolates and identified ST8 as the most prevalent multi-locus sequence type (MLST), representing 242 isolates (53.4%) (Fig 1B). Among the 128 BJI episodes that involved multiple isolates, 14 had >1 ISLs present using a single nucleotide polymorphism (SNP) of >100 difference. Applying the same cutoff to the 85 patients with multiple episodes, 64.7% involved the same ISL. In total, 35 patients (22%) had >1 ISL (Fig 1C).

**Conclusion:**

We found significant diversity among MRSA strains within patients, across infection episodes or anatomic sites. These findings highlight the need for comprehensive genomic investigation of BJIs to better understand the dynamics of MRSA infections and improve prevention strategies.

**Disclosures:**

All Authors: No reported disclosures

